# Hyperlipidemia and hyper glycaemia in Breast Cancer Patients is related to disease stage

**DOI:** 10.12669/pjms.341.14841

**Published:** 2018

**Authors:** Uzma Raza, Mahay Rookh Asif, Asif Bin Rehman, Aminuddin Sheikh

**Affiliations:** 1 Dr. Uzma Raza, M.Sc, MPhil, Ph.D. Professor and HOD of Biochemistry, Hamdard University, Karachi, Pakistan; 2 Dr. Mahay Rookh Asif, MBBS, Ph.D. Professor of Pharmacology & Therapeutics, Dow International Medical College, Dow University of Health Sciences, Karachi, Pakistan; 3 Dr. Asif Bin Rehman, MBBS, Ph.D. Professor of Pharmacology, Hamdard College of Medicine & Dentistry, Hamdard University, Karachi, Pakistan; 4 Dr. Aminuddin Sheikh, MBBS, M.Phil. Professor of Pathology, Hamdard College of Medicine & Dentistry, Hamdard University, Karachi, Pakistan

**Keywords:** Breast Neoplasms, Lipid Profile, Pathology

## Abstract

**Objective:**

The study was designed to determine the hyperlipidemia in breast cancer of patients at disease presentation, without any treatment and to correlate these variations with disease stage.

**Methods:**

This cross sectional study was conducted at Liaquat National teaching hospital in Karachi from 2006 to 2011, Age and family history of 208 breast cancer patients with infiltrating Ductal Carcinoma were compared with 176 matched control subjects. Married females were selected, with children and short breast feeding period. Cancer stage I-III was considered for the study and patients were grouped on the basis of Tumor grade, Tumor size, lymph node metastasis and disease free survival. Disease staging was based on tumor size and lymph node metastasis. Biochemical estimations included variations in random blood glucose level and lipid profile.

**Results:**

Lipid profile and random blood glucose level were found significantly high (p<0.05) compared to control subjects. Hyperlipidemia was significantly high in breast cancer patients with lymph node metastasis. On increase in tumor grade I to II, increase in total cholesterol (4%), LDL-cholesterol 23% and 11% increase in triglycerides was observed. On Tumor size increase from ≤2 to 2.5cm, increase observed in blood random glucose level was (4%), total cholesterol (1.7%) triglycerides (2%) and LDL (3%) whereas HDL was (2%) low. These variations remain insignificant on further increase in tumor size and grade.

**Conclusion:**

Study suggests that variation in lipid profile and blood random glucose level is associated with disease stage. No independent correlation of hyperlipidemia and hyperglycemia was developed with disease free survival.

## INTRODUCTION

Lipids have always been held responsible for pathogenesis of cardiovascular diseases. Researchers have recently discovered the association of circulating lipids with diseases including breast cancer.^1^ In Pakistan incidence of occurrence is 2–5 times higher than other countries and one out of nine females suffer from this disease.^2^ Breast cancer occurrence related hyperlipidemia was related with short breast feeding period, family breast cancer history due to mutation in BRCA1 and BRCA2.^3^ Obesity with high BMI was related with ER positive breast cancer irrespective of menopausal status.^4^ Infiltrating ductal carcinoma is an invasive type of breast cancer arising from the duct and infiltrate into the surrounding tissues.^5^ Cancer stages are I-III and IV, stages I-III define cancer spread with increase in tumor size and lymph node involvement whereas stage IV is related with cancer spread to other parts of the body.^6^ Tumor Grade has specific significance in cancer biology, high Tumor Grade was associated with micro calcification, necrosis and fibrosis.^7^ One of the main prognostic factors that determine cancer prognosis is tumor size. Large tumor size is associated with lymph node metastasis.^8^ It was reported that breast cancer occurrence in women initiate a number of biochemical changes. Peripheral tissues provide free fatty acids to enhance tumor growth.^9^ Less glucose consumption was reported by nonmetastatic cells as compared to metastatic cells, therefore metastatic cells consume more glucose and express high glycolysis.^10^

The aim of the study was to evaluate the hyperglycemia and hyperlipidemia including total cholesterol, triglycerides, LDL-cholesterol and HDL-cholesterol in breast Cancer Patients at initial diagnosis and to correlate the estimated changes with Tumor histopathology.

This study will create awareness in females to safe themselves from this deadly disease by controlling their blood sugar levels and lipid profile through changes in dietary habits and life style.

## METHODS

The study was conducted at Liaquat National Hospital Karachi on 208 non-pregnant female breast cancer patients of 30-60 years age, having children with breast feeding period less than six months. Females with past disease history other than cancer or on any medication were excluded from the study. Patients selected had primary infiltrating ductal carcinoma in one breast only. Disease stage I-III was included and stage IV patients (cancer metastasis to other organs) were excluded from the study on the basis of bone scan and ultra sound. Life history and social status of 176 healthy control subjects was matched with that of breast cancer patients.

The tumor Histopathology considered for the study was Tumor Grade-I to III, Tumor sizes < 2 cms, 2-5 cm and > 5 cm. Patients were classified on the basis of Axillary lymph node (ALN) metastasis. Seventy six Patients were without lymph Node metastasis and 148 patients with ALN metastasis. Cancer staging was according to the TNM system, stage IA (invasive tumor < 20 mm. ALN without tumor), Stage IB (tumor < 20 mm. metastasis in ALN 0.2- 2mm, prognostic group(PG)- 2), stage IIA tumor 20-50mm ALN metastasis free, PG- 3), IIB (tumor 20-50mm 1-3 ALN metastasis prognostic group 1/ tumor > 50mm no tumor in ALN. PG 2), stage IIIA (Invasive tumor > 50mm, cancer in 4-9 ALN, PG 4).^11^

Blood samples were collected at the time of disease diagnosis, without any treatment. Tissue histopathology was performed according to method of Harris's Haematoxylin and Eosin staining method.^12^ Nottingham Modification of the Bloom-Richardson system was used for microscopic grading of breast carcinoma.^13^ The biochemical estimations were done by Kit provided by Roche Diagnostics GmbH, D-68298 Mannheim, Germany. Chemistry analyzer Hitachi 912 used for analysis was provided by Roche diagnostic Basil Germany. Schmidt and Peterson and Young method was used for estimation of glucose, as a reference.^14^ Estimation of Cholesterol method was based on Tinder reaction,^15^ HDL was estimated by polyethylene glycol modified enzymes and dextran sulfate.^16^ LDL was estimated by modified method of Friedewald 1972.^17^ Triglycerides were detected by Wahlefeld's method modified in 1990.^18^

All experimental data was expressed as mean ± SEM. Data was analyzed by one way ANOVA using SPSS version (version 19.0). p< 0.05 was considered as significant.

## RESULTS

In all breast cancer patients (208) blood random glucose level, total cholesterol (TC), triglyceride (TG) and low density lipoprotein (LDL) were significantly high and high density lipoprotein (HDL) was found significantly low as compared to control subjects, *p<0.05. Level of blood random glucose and lipid profile was significantly high in patients with lymph node metastasis as compared to that without lymph node metastasis ^▲^p<0.05 (Table-I).

**Table-I:** Blood random glucose level and lipid profile in breast cancer patients.

Blood random glucose and lipid profile level (mg/dL)	Patients with lymph node metastasis	Patients without lymph node metastasis	Control Subjects
Blood Glucose	135.0 ± 0.27 *^▲^	132.1 ± 0.45 *	129.7 ± 0.22
Cholesterol	190.9 ± 0.56 *^▲^	183.3 ± 0.80	180.9 ± 0.57
Triglyceride	134.0 ± 0.66 *^▲^	118.1 ± 1.04 *	105.7 ± 0.41
LDL	118.9 ± 0.64 *^▲^	103.0 ± 1.23 *	89.5 ± 0.28
HDL	40.0 ± 0.27 *^▲^	52.3 ± 0.88 *	54.0 ± 0.23

* p<0.05, compared with the normal control group.

▲ p<0.05 in patients with lymph node metastasis compared with those without lymph node metastasis.

Patients were grouped on the basis of tumor histopathology including tumor grade, tumor size and ALN metastasis status. Tumor Grade-I patients were 32 out of these 62.5 % (20) patients were without ALN metastasis. 105 patients were with tumor Grade-II, out of these 26.7% (28) were without ALN metastasis. Patients with tumor Grade-III were 71 out of these 16.9% (12) were without ALN metastasis, percentage of patients without axillary lymph node (ALN) metastasis decreased with the increase in tumor grade. Patients with ALN metastasis in all tumor grades had significantly high Blood random glucose level, TC, TG and LDL whereas HDL was found significantly low as compared to the patients without ALN metastasis, *p<0.05. In patient with ALN metastasis and tumor Grade-II (77), 4% increase in total cholesterol, 23% increase LDLcholesterol and 11% increase in triglycerides were observed as compared to 12 tumor Grade-I patients. Among patients without ALN metastasis increase in Lipid profile percentage was less in 28 tumor Grade-II patients as compared to 20 Grade-I patients, total cholesterol and triglyceride increased 2% and LDL-cholesterol 20%. Percentage variation in glucose level was not observed on increase in tumor grade. As tumor Grade-Increased from II to Grade-III 1.7% increase in TC of 59 patients was observed whereas in 12 patients without ALN metastasis increase in cholesterol was 1.5% and TG 10.9% (Table-II).

**Table-II:** Effect of tumor grade on blood glucose level and lipid profile in breast cancer patients.

Blood random glucose and lipid profile level (mg/dL)	Tumor Grade-I		Tumor Grade-II		Tumor Grade 3	

	With Metastasis	Without Metastasis	With Metastasis	Without Metastasis	With Metastasis	Without Metastasis
Blood Random Glucose	134.7± 0.99*	130.1± 0.42	134.7± 0.37*	132.7± 0.71	135.5 ± 3.33	134.0± 0.98
Cholesterol	184.1 ± 0.89*	180.4± 0.15	191.6± 0.69*	183.8 ± 1.28	191.4 ± 0.96*	186.7 ± 2.05
HDL	41.3 ± 0.83*	53.6 ± 0.64	39.4 ± 0.36*	52.0 ± 1.78	38.3 ± 0.35*	50.7± 0.88
LDL	97.3± 0.96*	91.9± 0.91	120.6 ±0.44*	110.3 ± 1.15	121.1 ± 0.64*	104.1 ± 0.83
Triglycerides	120.2 ± 1.76*	114.2± 1.31	134.2± 0.71*	116.2 ± 1.32	136.5 ± 0.99*	128.9± 0.98

* p<0.05 as compared with the patients without lymph node metastasis

Effect of disease stage based on tumor size and ALN on random blood glucose level and lipid profile was evaluated in breast cancer patients. Total 16 patients were studied with tumor size < 2cm, out of these 13 patients were without ALN metastasis (81%) at cancer stage IA and 03 patients with ALN metastasis (19%) were at cancer stage IB. No comparative significant change was found in blood random glucose, total cholesterol and LDL levels. High density lipoproteins were significantly low and triglycerides were significantly high in patients with metastasis as compared to that without metastasis. Patient group of 76 was studied with tumor size 2-5 cm, 27 patients (35.5%) were without ALN metastasis, at cancer stage IIA and 49 (64.4%) with ALN metastasis at cancer stage IIB. Patients at cancer stage IIB had significantly high blood sugar, TC, HDL, LDL levels and significantly low HDL levels as compared to the patients at cancer stage IIA. Total 116 Patients with tumor size > 5cm were studied, 96 patients (80.2%) with ALN metastasis at cancer stage IIIA and 20 patients (17.2%) without ALN metastasis at cancer stage IIB. Patients with cancer stage IIIA had significantly high blood random glucose level, TC, TG and LDL whereas significantly low HDL as compared to the patients at cancer stage IIB *p<0.05. Results suggest that biochemical variations are increased with the disease advancement accompanied by tumor size increase from <2 to 2,5cm. and ALN metastasis. Blood random glucose level (4%), TC (1.7%), TG (2%) and LDL (3%) increased whereas HDL significantly decreased (2%), these variations remain unchanged on further increase in tumor size (2.5- 5cm). In patients without ALN metastasis biochemical variations with the increase in tumor size were less, increase was found only in TC (2%), LDL-cholesterol (6.5%) and TG (5.2%) (Table-III).

**Table-III:** Effect of Cancer stage on blood glucose level and lipid profile in breast cancer patients.(11)

	Tumor size < 2cm		Tumor size (2-5)cm		Tumor size > 5cm	

Blood random glucose and lipid profile level (mg/dL)	Without Metastasis Stage IA	With Metastasis Stage IB Prognostic Group (2)	Without Metastasis Stage IIA Prognostic Group (3)	With Metastasis Stage IIB Prognostic Group (1)	Without Metastasis Stage IIB Prognostic Group (2)	With Metastasis Stage IIIA Prognostic Group (4)
Blood random Glucose	131.7± 0.88	129.7±0.88	131.8± 0.68	135.4± 0.42*	132.7±0.81	135.0±0.34*
Cholesterol	179.8± 0.82	187.7± 4.98	183.3± 1.14	190.9± 1.05*	185.5±1.64	191.0±0.66*
HDL	51.8±1.10	40.9±1.53*	51.9 ± 1.51	40.0± 0.41*	53.3 ±1.55	39.5±0.34*
LDL	97.5± 2.73	111.0±6.12	103.9± 2.05	114.6± 1.48*	105.3±1.42	121.4±0.43*
Triglycerides	113.0±1.64	133.7±3.85*	118.9± 1.61	132.2± 1.47*	120.4±1.73	135.0±0.66*

* p<0.05 as compared with the patients without lymph node metastasis.

Disease free survival (DFS) was the period between treatment completion and reappearance of first disease symptom and it was calculated in months. About 18.75% (39) patients had DFS < 24 months, out of these 79.5% (31) were patients with ALN metastasis. 74% (154) patients had DFS of 24-48 months, patients with lymph node metastasis were 71% (110). 72% (15) patients had DFS of > 48 month and 46% (07) were with ALN metastasis. ALN metastasis is associated with cancer stage IB, IIB and IIIA, study indicates low percentage of patient with progressed disease is associated with high DFS. In patients with ALN metastasis blood random glucose level, total cholesterol, LDL and triglyceride were significantly high and HDL was significantly low in patients as compared to the patients without ALN metastasis*p<0.05 (Table-IV). No relation of biochemical variation was developed with DFS.

**Table-IV:** Effect of blood random glucose level and lipid profile on disease free survival in breast cancer patients.

	Months< 24		Months 24-47		Months>48	

	With Metastasis	Without Metastasis	With Metastasis	Without Metastasis	With Metastasis	Without Metastasis
Blood random Glucose	135.4 ± 0.61*	132.1 ± 1.18	134.9± 0.31*	132.2 ± 0.53	134.7 ± 0.94*	131.4 ± 1.30
Cholesterol	190.2 ± 1.15*	183.1 ± 2.93	190.9 ± 0.66*	183.4 ± 0.93	193.8 ± 2.11*	182.9 ± 2.06
HDL	40.3 ± 0.36*	49.4 ± 1.67	39.9 ± 0.34*	53.0 ± 1.10	40.1 ± 0.42*	51.4 ± 1.86
LDL	119.7 ± 0.81*	103.2 ± 3.73	118.4 ± 0.82*	102.2 ± 1.36	122.4 ± 0.99*	106.9 ± 3.82
Triglycerides	135.2 ± 1.68*	113.0 ± 2.09	133.7 ± 0.74*	119.2 ± 1.24	132.8 ± 1.03*	115.4± 2.73

* p<0.05 as compared with the patients without lymph node metastasis.

## DISCUSSION

In the breast cancer patients, significant variations were observed in random blood glucose, and lipid profile as compared to control subjects, *p<0.05 their role in disease occurrence may be predicted. Hyperlipidemia and hyperglycemia were comparatively more significant in patients with lymph node metastasis. Lymph node status indicates the ability of tumor to spread.^6^ It was suggested by the studies that increased plasma cholesterol level plays a role in breast cancer occurrence and progress.^19^

The study was further extended to evaluate the relation between biochemical changes and disease stage based on histopathology, including tumor grade, tumor size and lymph node metastasis. Past Studies have correlated high tumor grade with lymph node metastasis.^20^ Patients with lymph node metastasis in all tumor grades had significantly high blood random glucose level and hyperlipidemia percentage as compared to patients without metastasis, indicating tumor grade along with lymph node metastasis was responsible for increasing lipid profile. Present study indicated significant blood glucose and lipid profile increase in patients with lymph node metastasis at disease stage (IB, IIB, IIIA) as compared to that without lymph node metastasis with increase in tumor size (IA, IIA). The cancer mass present in the lymph nodes increased the total cancer mass from which the spread can take place. Cancer spread can be explained by the process including (size + node equation).^21^ Percentage variation in blood glucose and lipid profile were high in patients up to tumor size 2.5cm, variations were less if the tumor size increased beyond 5cm. Therefore, study correlates the biochemical variations with tumor activity at early stage. Alterations in lipid profile levels have been significantly related with disease stage.^22^

Disease free survival (DFS) of the patients were correlated with biochemical variations. Hyperlipidemia and hyperglycemia were more significant in patients with lymph node metastasis as compared to patients without lymph node metastasis. About 79% patients with lymph node metastasis had DFS less than 24 months, 71.4% patients had DFS OF 24-48 months and 46% metastatic patients had DFS of greater than 48 months. No relation between hyperlipidemia and DFS was developed. Mortality risk by breast cancer was related with lymph node metastasis earlier.^23^ Hyperglycemia in breast cancer patients has been linked with insulin resistance. Insulin exerts various metabolic effects and also works in the proliferation of cells. Tumor growth can be enhanced by hyperglycemia since DNA synthesis of tumor cells is increased by hyperglycemia.^24^

Breast cancer occurrence is related with estrogen and estrogen increased the growth hormone release which in turn induces a state of insulin resistance.^25^ Alterations in lipid profile related to histopathology may be attributed to the metabolic changes produced by tumor activity.

Risk of breast cancer reoccurrence has been correlated with, overall hyperlipidemia including high serum cholesterol, LDL-cholesterol and triglycerides.^26^ According to the past study low level of HDL and high level of LDL-cholesterol promotes metastasis & proliferation.^27^ The increased level of triglycerides may be attributed to the decreased hepatic triglyceride lipase activity.^28^

### Strength and Limitations

Study indicates that tumor with poor histopathology including lymph node metastasis, high tumor grade and large tumor size had significant biochemical variations and low disease free survival. Limitation was that samples were collected from one center only.

## CONCLUSION

Study related hyperlipidemia and hyperglycemia with disease stage and was significant in patients with progressed disease IB, IIB and IIIA. No independent relation of disease free survival was developed with hyperlipidemia and hyper glycemia. Disease free survival was dependent on tumor histopathology.

### Implications

Study can guide the oncologist to predict the disease prognosis on the basis of pretreatment biochemical profile.

### Authors' Contribution

***UR:*** Main author, conceived research idea, data collection & initial compilation of manuscript.

***MRA:*** Manuscript review.

***ABR:*** Literature review.

***AS:*** Guided in the capacity of Histopathologist.

## References

[1] Hasija K, Bagga HK (2005). Alterations of serum cholesterol and serum lipoprotein in breast cancer of women. Indian J Clin Biochem.

[2] Asif HM, Sultana S, Akhtar N, Rehman JU, Rehman (2014). Prevalence, risk factors & disease knowledge of breast Cancer in Pakistan. Asian Pac J Cancer Prev.

[3] Laisupasain P, Thompat W, Sukarayodhin S, Somprom A, Sudjaroen Y (2013). Comparison of Serum lipid profiles between normal control and breast cancer patients. J Lab Physician.

[4] Tamaki K, Tamaki N, Terukina S, Kamada Y, Uehara K, Arakaki M (2014). The correlation between body mass index and breast cancer risk or estrogen receptor status in Okinawan women. Tohoku J Exp Med.

[5] Michael JG, Peter J.M, Ronald A.M (1994). Cancer of the breast. Oxford Textbook of surgery.

[6] (2014). HealthLink(BC Government) Cancer staging and Grading Accessed January 3.

[7] Webster LR, Bilous AM, Willis L, Byth K, Burgemeister FC, Salisbury EL (2005). Histopthologic indicators of breast cancer biology: insights from population mammographic screening. Br J Cancer.

[8] Ke-Da Y, Jiang YZ, Chen S, Cao ZG, Wu J, Shen ZZ (2012). Effect of Large tumor size on cancer Specific Mortality in Node negative Breast cancer. Myoclin Proc.

[9] Quevedo-Coli S, Crespi C, Benito E, Palou A, Roca P (1997). Alterations in circulating fatty acids and the compartmentation of selected metabolites in women with breast cancer. Biochem Mol Biol Int.

[10] Robey IF, Lien AD, Welsh SJ, Baggett BK, Gillies RJ (2005). Hypoxia-inducible factor- 1 alpha and the glycolytic phenotype in tumours. Neoplasia.

[11] Breast Cancer: Stages Approved by Cancer. Net Editorial Board 04/2017 Original source AJCC Cancer staging manual.

[12] Ramnik S (2006). Histopathology: In text book of medical Laboratory Technology.

[13] John DB, Marilyn G (2002). Breast. Theory and Practice of histological techniques.

[14] Schmidt FH (1961). Enzymatic determination of glucose and fructose simultaneously. Klin Wschr.

[15] Trinder P (1969). Enzymatic calorimetric method CHOD – PAP. Ann Clin Biochem.

[16] Sugiuchi H, UJi Y, Okabe H, Irie T, Uekama K, Kayahara N (1995). Direct measurement of high-density lipoprotein cholesterol in serum with polyethylene Glycol – modified enzymes and sulfated α– cyclodextrin. Clin Chem.

[17] Friedewald WF, Levy RI, Frederickson DS (1972). Estimation of LDL-cholesterol concentration without use of the preparative ultracentrifuge. Clin Chem.

[18] Shephard MDS, Whiting Falsely MJ (1990). Estimation of triglyceride in lipemic plasma by the enzymatic Triglyceride method with modified Trinderschromogen. Clinchem.

[19] Llaverias G, Danilo C, Mercier I, Daumer K, Capozza F, Williams TM (2011). Role of Cholestrol in development and progression of breast cancer. Am J Pathol.

[20] Saiz E, Toonkel R, Poppiti RJ, Robinson MJ (1999). Infiltrating breast carcinoma smaller than 0.5 centimeters: is lymph node dissection necessary?. Cancer.

[21] Michaelson JS, Chen LL, Silverstein MJ, Cheongsiatmoy JA, Mihm MC, Sober AJ (2009). Why cancer at the primary site and in the lymph nodes contributes to the risk of cancer death. Cancer.

[22] Shah F D, Shukla S N, Shah P M (2008). Significance of alterations in plasma lipid profile levels in breast cancer. Interg Cancer Ther.

[23] Michaelson JS, Chen LL, Silverstein MJ, Mihm MC, Sober AJ, Tanabe KK (2009). How cancer at the primary site and in the lymph nodes contributes to the risk of cancer death. Cancer.

[24] Saba Z, Ujpal M (2006). Correlation of insulin resistance and neoplasms. Magy Onkol.

[25] Laisupasin P, Thompat W, SukaraYodhin S, Sornprom A, Sudiaroen Y (2013). Comparison of serum lipid profile between normal control & breast cancer patients. Lab Physicians.

[26] Alexopoulos CG, Blatsios B, Avgerinos A (1987). Serum lipids and lipoprotein disorders in cancer patients. Cancer.

[27] Santos Dos CR, Matias Ines GT, Matostion J, Fonseca I, De Almeida JM, Dias S (2014). LDLCholesterol signaling induces Brest Cancer Proliferation and invasion. Lipid Health Disease Published Online.

[28] Taira M, Takasu N, Komiya I (1998). Severe hypertriglyceridemia induced by tamoxifen. Nippon Ronen Igakkai Zasshi.

